# An Overview of Carbon Nanotubes and Graphene for Biosensing Applications

**DOI:** 10.1007/s40820-017-0128-6

**Published:** 2017-02-07

**Authors:** Zanzan Zhu

**Affiliations:** National Cancer Centre Singapore, 11 Hospital Drive, Singapore, 169610 Singapore

**Keywords:** Biosensor, Carbon nanotubes (CNTs), Graphene

## Abstract

With the development of carbon nanomaterials in recent years, there has been an explosion of interests in using carbon nanotubes (CNTs) and graphene for developing new biosensors. It is believed that employing CNTs and graphene as sensor components can make sensors more reliable, accurate, and fast due to their remarkable properties. Depending on the types of target molecular, different strategies can be applied to design sensor device. This review article summarized the important progress in developing CNT- and graphene-based electrochemical biosensors, field-effect transistor biosensors, and optical biosensors. Although CNTs and graphene have led to some groundbreaking discoveries, challenges are still remained and the state-of-the-art sensors are far from a practical application. As a conclusion, future effort has to be made through an interdisciplinary platform, including materials science, biology, and electric engineering.

## Introduction

A biosensor refers to a sensing device that transfers a biological event to a measurable signal. It usually consists of a biological recognition element and a transducer to translate the biology information to the measurable signal [1]. To be specific, the biological recognition element in a biosensor must be selective to a certain biomolecule, biology process, or chemical reaction. Depending on the types of the recognition elements, the biosensors are able to detect a wide range of biological elements, such as nucleic acids, antibodies, enzymes, bacteria, and viruses [2]. To date, the biosensors have been tested for their usage in food, environmental, and human samples [3]. The biological recognition elements often are immobilized onto the surface of transducer with high bioactive for targeting. The attachment methods include adsorption, encapsulation, entrapment, covalent binding, and cross-linking [4]. The interaction between the recognition element and the target will then be monitored and further converted to a readable signal like current. Depending on the interaction models, different types of transducers can be utilized to convert the recognition events into a digital signal that is proportional to the presence and the amount of the target. The most common transducing methods include electrochemical, optical, piezoelectric, and magnetic. Among them, electrochemical, electrical, and optical techniques are very popular due to the fast response and the flexibility in employing recognition elements [5].

A great effort has been given in the past few years in the worldwide range on developing novel biosensors with high sensitivity and selectivity. The recent, fast development of nanomaterials has made a profound influence on the development of biosensors. The application of nanomaterials has been given to all technical components of biosensors from recognition components to signal processers. When the material’s size is reduced to nanoscale, the interesting changes in chemical and physical properties are happened due to two principal factors: surface effect and quantum effect. The surface to volume ratio of nanomaterials increases dramatically compared to their bulk form and is able to improve the sensitivity of biosensors through increasing the interface for recognition element allocation. The quantum confinement phenomenon can lead to an increase in the band-gap energy and a blue shift in light emission with decreasing size. As a result, the electrical and optical properties of nanomaterials become size and shape dependent. These essential features of nanomaterials make it possible to turn chemical and physical properties to specific biosensor applications by controlling their size, shape, and chemical composition [5].

The world of nanomaterials is huge and consists of various materials with different nature, size, shape, composition, chemistry, etc. For biosensors, nanomaterials like carbon nanotubes (CNTs) and graphene are popular and at the forefront of the research [6, 7]. These two are also the most representatives in the big family of carbon nanomaterials [8]. To date, CNTs and graphene have been widely studied for biosensor applications due to their unique chemical and physical properties [9]. In this review article, we give a brief review on the recent developments of CNT- and graphene-based biosensors, aiming to provide a comprehensive introduction to researchers who are new to this field. The article also gives a brief perspective summary on the challenges of these biosensors toward the practical application.

## Carbon Nanotube-Based Biosensors

CNTs have had a profound impact on a wide range of applications because of their unique electronic, chemical, and mechanical properties [10]. CNTs are made of cylinders of *sp* ^2^-hybridized carbon atoms with several nanometers in diameter and many microns in length. There are two classes of CNTs, single walled carbon nanotubes (SWNTs) and multiwalled carbon nanotubes (MWNTs). SWNTs can be considered as one rolled-up graphene sheet, while MWNTs are concentric tubes separated by about 0.34 nm of two or more rolled-up graphene sheets. SWNTs have very unique electrical properties, depending on the chirality of the wrap, and they can behave as either metals or semiconductors [11, 12]. Recent studies have established the fact that several intriguing properties of CNTs, such as their nanodimensions and graphitic surface chemistry [13], make them extremely attractive for new types of electrochemical, electric, and optical biosensors [9].

### Pre-functionalization of CNTs

It is known that one of the biggest barriers for developing CNTs-based biosensors is the dispersion issue caused by the high surface energy of CNTs. It results in difficulties in handling CNTs in a controlled way, and most solvents cannot suspend CNTs well. In order to overcome this deficiency, CNTs are usually functionalized with polymer and small molecules to render the surface compatibility to solvents and bioenvironments for further biosensing applications [14]. Surface functionalization can be made through covalent and noncovalent bonding. For covalent bonding approach, the most common one is the oxidation of CNTs with an acid such as nitric acid and the mixture of nitric acid and sulfuric acid [15]. Depending on the types of the employed oxidizing agents, carboxyl or hydroxyl groups are introduced onto the ends and the sidewalls of CNTs during the oxidation [16]. These groups lead the reduction of the van der Waals interactions between CNTs and enable further modifications to covalently connect with other molecules, like long alkyl chains, polymeric molecules, dendrimers, nucleic acids, and enzymes [17]. Compared to the oxidation approach, the direct covalent functionalization gives stronger influence on chemical and physical properties of CNTs and provides opportunities for further CNTs-associated applications [17]. In nonplanar *π*-conjugated carbon framework, two factors govern the chemical reactivity of the sidewalls of CNTs: (1) curvature-induced pyramidalization at the individual carbon atoms and (2) misalignment of *π*-orbital between adjacent carbon atoms [18–20]. Some highly reactive species (like halogens, radicals, carbenes, or nitrenes) are the ideal reagents for covalent functionalization of the sidewalls [18]. These groups can be bonded onto *π*-conjugated carbon structures of the CNTs through a series of addition reactions as introduced in the article by Balasubramanian and Burghard [17].

Modification via 1, 3-dipolar cycloaddition is another widely used type of covalent sidewall functionalization of CNTs [21]. The attachment of 1, 3-dipolar cycloaddition of azomethine-ylide onto the graphite sidewall of CNTs is generated by condensation of an aldehydes and an α-amino acids [22]. A pyrrolidine ring was formed on the CNTs surface through the reaction between C=C bond and azomethine-ylide [21]. Functional groups introduced via above methods enable CNTs soluble in aqueous or organic solvents and open the possibility for the further fabrication of CNT-based biosensors [14].

Compared to covalent functionalization, noncovalent functionalization of CNTs keeps the structure of CNTs intact and thus retains their physical properties [23]. Noncovalent interactions include electrostatic interaction, *π*–*π* stacking, van der Waals force, and hydrophobic or hydrophilic interactions are efficient methods for the immobilization of biomolecules onto CNTs surface [23, 24]. Chen et al. reported the noncovalent functionalization of CNTs with certain aromatic molecules through *π–π* stacking [25]. A biofunctional molecule, 1-pyrenebutanoic acid, succinimidyl ester was found to strongly interact with the basal plane of graphite on the sidewall of SWNTs via *π*–*π* stacking. The anchored succinimidyl ester on the CNTs surface could be used to attach DNA or proteins through the formation of amide bonds [25]. Some other biochemical active molecular with amine groups, such as streptavidin and ferritin, has been immobilized onto CNTs using above approach as well [25, 26]. Similarly, many biocompatible polymers can be wrapped or physically adsorbed onto the surface of CNTs by *π–π* stacking. O’Connell et al. [27] wrapped SWNTs with polystyrene sulfonate (PSS) and polyvinyl pyrrolidone (PVP) to render them reversible solubility in water. Furthermore, fluorescein-polyethylene glycol (Fluor-PEG) has been found able to be attached onto SWNTs through strong *π–π* interactions by Nakayama-Ratchford et al. The finite fluorescence intensity of fluorescein-PEG/SWNTs can be used in biosensor and biomedical imaging [28]. Chitosan (CHI), as a biopolymer with good film-forming ability, has been widely used in the detection of various biological molecules through the formation of a special CHI–CNTs system [29]. Using surfactants to wrap around the surface of CNTs is another strategy to noncovalently modify CNTs. Water-soluble surfactants, like sodium dodecyl sulfate (SDS) and cetyltrimethylammonium bromide (CTAB), can be applied to improve solubility and stability of CNTs in various suspensions [30].

### CNT-Based Electrochemical Biosensors

Electrochemical biosensor is a two- or three-electrodes electrochemical cell, which can transfer a biological event to electrochemical signal. They often contain a biological recognition element on the electrode which reacts with the analyte and then produce electrochemical signal [31]. CNT-based electrochemical biosensors play an important role in CNT-based biosensors because of their intrinsic advantages such as high sensitivity, fast response, easy operation, and favorable portability. Based on the method of the recognition process, CNT-based electrochemical biosensors can be divided into biocatalytic sensors and bioaffinity sensors. Biocatalytic sensors use the biological recognition element (e.g., enzyme) that can produce electroactive species, while bioaffinity sensors monitor a binding event between the biological recognition element and the analyte [32, 33]. CNT-based enzymatic electrochemical biosensors and CNT-based bioaffinity electrochemical sensors will be reviewed in details.

#### CNT-Based Enzymatic Electrochemical Biosensors

Enzymatic biosensors that combine electrochemical technology with specificity of enzyme have provided great opportunities for strategies in the early diagnosis [34]. The direct electron transfer between the redox-active center of enzyme and the electrode without mediators is critical to the development of enzymatic biosensor. However, because the active centers of enzymes are surrounded by a thick protein layer and located deeply in hydrophobic cavity of molecules, the direct electrochemistry of enzyme is very difficult [35, 36]. Therefore, the use of an electrical connector is required to enhance the transportation of electrons. CNTs, with their small size, extraordinary electrochemical properties, and high specific surface area, have been widely used to promote electron transfer between the electrode and the redox center of enzyme [6]. During the past few years, there have been many reports of CNT-based enzymatic biosensor for the detection of clinically important analytes through the electrochemical reactions catalyzed by various enzymes [6], such as glucose oxidase (GOx) [37], horse radish peroxidase (HRP) [38], lactate oxide [39], malate dehydrogenase (MDH) [35], and so on. One of the major challenges for the design of CNT-based enzymatic biosensor is how to achieve stable attachment of enzyme while still retaining their bioactivity. According to the different architectures, there are four main types of CNT-derived enzyme electrodes as discussed in the following.

##### CNT Paste Enzyme Electrodes

The first application of CNTs as electrode was reported by Britto et al. [40]. A carbon nanotube paste electrode (CNPE) was constructed by using bromoform as binder to mix with carbon nanotubes, and better performance of electrochemical oxidation toward dopamine was observed on CNPE than other carbon electrodes [40]. In a similar manner, CNTs have been mixed with mineral oil for glucose detection by adding GOx into the composite material. A detection limit of 0.6 mM was obtained with the CNPE containing 10 wt% GOx [41].

##### CNT-Modified Electrodes with Immobilized Enzymes

In most cases, CNT-based enzymatic biosensors were fabricated by modifying electrodes with CNTs and enzymes via different approaches [34]. Similar to the functionalization of CNTs, methods for linking enzyme onto CNTs include noncovalent and covalent interaction. Noncovalent approach can preserve the structural integrity and properties of enzyme as well as provide high surface loading of enzyme [42]. However, the interaction between enzyme and CNTs is not strong; thus, the immobilized enzyme may be gradually lost during the use. This limitation can be overcome by adsorbing enzymes onto polymer or nanoparticles-modified CNTs. Cai and Chen [37] dispersed CNTs in the solution of CTAB and then mixed with graphene oxide (GO). Nafion was used as a binder to hold the GOx/CNTs mixture on the electrode. The promotion effect of CNTs on the direct transfer of glucose oxidase which immobilized on CNTs was observed. In our previous work, bamboo-shaped carbon nanotube/chitosan film has been used for the immobilization of horseradish peroxidase (HRP) and related bioelectrochemical studies. The results indicated that immobilized HRP in the film shows excellent bioelectrocatalytic activity toward H_2_O_2_ [43]. As a further example, a biosensor for glucose detection has been obtained by the deposition of Pt nanoparticles onto Nafion-containing GOx/CNTs film. The designed glucose biosensor achieved a fast response time of 3 s and a low detection limit of 0.5 µM [44].

**Scheme 1:**
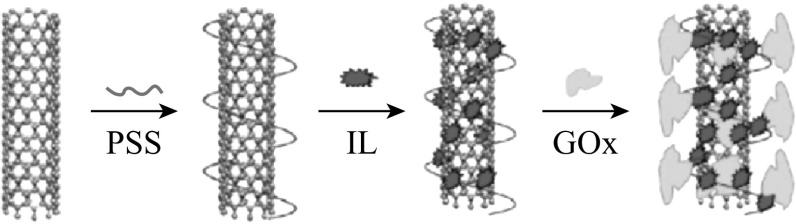
The immobilization of glucose oxidase (GOx) on the surface of SWNTs by using enzyme adsorption involves a layer-by-layer technique. *PSS* poly(sodium 4-styrenesulfonate), *IL* ionic liquid. Reprinted with permission from Ref. [45]. Copyright (2009) American Chemical Society

Another avenue for enzyme adsorption involves the layer-by-layer technique. For example, Wu et al. designed a glucose biosensor by assembling ionic liquids and GOx on poly(sodium 4-styrenesulfonate) (PSS)-coated CNTs surface through the electrostatic interaction (Scheme 1). They found that ionic liquids play an important role in affecting the electrocatalytic activity of GOx-IL-PSS-CNT/GC electrodes toward the oxidation of glucose [45]. In the work reported by Wang et al., negatively charged 11-mercaptoundecanoic acid (MUA) was initially modified on the gold electrode, following by the attachment of a positively charged redox polymer, poly[(vinylpyridine)Os(bipyridyl)_2_Cl^2+/3+^] and a GOx solution containing CNTs based on an electrostatic layer-by-layer (LBL) technique. It has been observed that the glucose electro-oxidation current increased 6–17 times compared to electrode without SWNTs. The sensitivity of the sensors could be controlled by tuning the number of layers [46].

Vertically aligned CNTs are another type of architecture for electrode modification. Vertically aligned CNTs coupled with enzyme on their tips facilitate rapid electron transfer compared to randomly distributed CNTs. It is because the CNTs tips have more activity sites than the sidewalls and the electrons directly transfer along the vertical direction of the tube [47]. Patolsky et al. reported a structural alignment of GOx onto the edge of CNTs that are linked to a gold electrode surface. Flavin adenine dinucleotide (FAD) was first covalently attached onto the edge of CNTs, and then GOx was electrically linked onto the immobilized FAD. The CNTs were used as electrical connectors between the enzyme redox and the electrode. The electrons are transported along distances greater than 150 nm, and the rate of electron transport is controlled by the length of the CNTs [48].

Compared with noncovalent enzyme adsorption, covalent conjugation provides durable attachment to prevent enzyme leakage. Ruhal et al. designed an amperometric malic acid biosensor by covalently immobilizing malate dehydrogenase (MDH) on MWNT-coated screen-printed carbon electrode using standard water-soluble coupling agents 1-ethyl-3-(3-dimethylaminopropyl)carbodiimide (EDC) and *N*-hydroxy-sulfo-succinimide (sulfo-NHS). The detection limit of malic acid was 60–120 µM, and the response time was 60 s [49]. In the work reported by Yu et al., vertically aligned SWNTs were initially assembled on ordinary pyrolytic graphite electrodes. Iron heme proteins horse heart myoglobin (Mb) and HRP were covalently attached onto the ends of the SWNTs via amide linkages, respectively. The detection limits toward H_2_O_2_ were found to be 70 nM for SWNT/Mb and 50 nM for SWNT/HRP. The authors suggested that vertically aligned SWNTs behaved electrically similar to a metal, conducting electrons from the external circuit to the redox sites of the enzymes [50].

##### CNT Forest Electrodes with Immobilized Enzymes

CNT forest electrodes refer to the use of vertically aligned carbon nanotube arrays as a sole conductive component instead of modifying it onto another electrode surface. In this case, a CNT array is grown directly on a substrate surface. Besides the general advantages of vertically aligned CNTs as mentioned above, the structure and morphology control of the tubes during the synthesis step provides more possibilities for diversifying the electrode design.

**Scheme 2:**
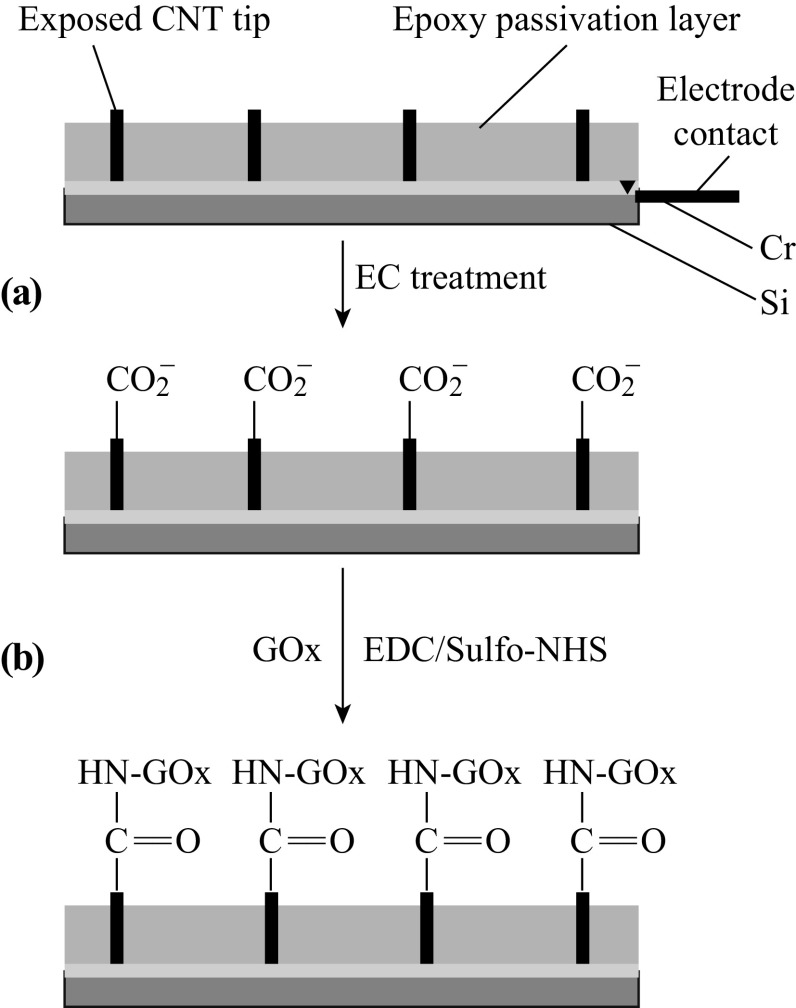
Fabrication of a glucose biosensor based on a CNT nanoelectrode: **a** electrochemical treatment of the CNT nanoelectrode assembly for functionalization. **b** Coupling of GOx to the CNT nanoelectrode ensembles. Reprinted with permission from Ref. [53]. Copyright (2004) American Chemical Society

Wang et al. developed a glucose biosensor based on gold/CNTs-GOx-modified electrode. CNT forest was grown on silicon substrate and then coated with a thin gold film. After the removal of the substrate, GOx was absorbed onto the Au/CNTs electrode. The designed glucose biosensor with electrode of Au/CNTs-GOx exhibits fast response and a low detection limit of 0.01 mM [51]. A cholesterol biosensor based on vertically aligned CNTs bioprobes on silicon substrates was developed by Roy et al. A Si substrate (2 × 5 mm^2^) with a layer of SiO_2_ (∼300 nm thick) was used as the platform. Electrodes consisting of Ti (100 nm)/Au (400 nm) were magnetron sputtered on the defined region. CNTs were grown on a window of 1 × 1 mm^2^ which was deposited by a Ni (∼10–30 nm)/Nb (∼200 nm) film. An insulated film was coated on the entire chip except for the region (1 × 1 mm^2^), through which the CNTs were grown. Before the immobilization of enzymes (cholesterol oxidase (ChO_x_)), cholesterol esterase (ChEs), and HRP onto CNTs, their surface was converted from hydropholic to hydrophilic through the surface modification with polyvinyl alcohol (PVA). A plot of the current response of the final CNTs sensor chip against cholesterol concentration can be found in a linear relationship observed in the range of 100–350 mg dL^−1^ of cholesterol concentration [52]. For covalent attachment of enzymes, Lin et al. reported a glucose biosensors based on CNTs nanoelectrode ensembles (NEEs). As shown in Scheme 2, aligned CNT arrays were grown on a Cr-coated Si substrate of 1 cm^2^ area, and GOx was then covalently attached onto CNT arrays through the formation of amide bond between their amine group and carboxylic acid group on the CNTs tips by using standard water-soluble coupling agents and sulfo-NHS. The limit of detection of the fabricated glucose biosensor based on an aligned CNTs NEE was found to be 0.08 mM [53].

**Scheme 3:**
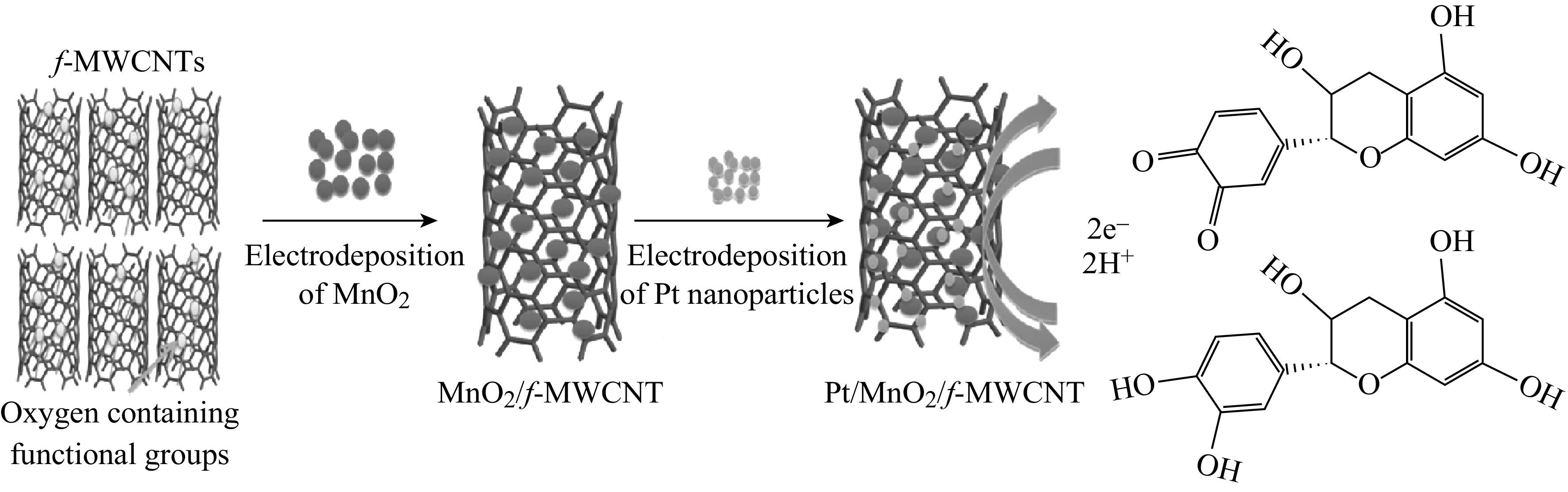
Illustration of the procedure used for the preparation of the Pt/MnO_2_/f-MWCNT film. Reprinted with permission from Ref. [54]. Copyright (2015) Royal Society of Chemistry

With the development of nanotechnologies in recent decades, nonenzymatic electrochemical biosensors have played an important role. Nonenzymatic biosensors, based on the oxidation of analyte catalyzed by electrocatalysts, avoid the usage of enzyme and can be considered as the future generation of electrochemical biosensor. Ezhil Vilian et al. reported a nonenzymatic biosensor for the determination of catechin using Pt nanoparticle-coated MnO_2_/CNTs nanocomposites. As shown in Scheme 3, the Pt/MnO_2_/f-MWCNTs used in this work were fabricated by successive electrodeposition of MnO_2_ and Pt nanoparticles onto CNTs surface. The nanocomposite-modified electrodes were employed to detect catechin and a low detection limit of ca. 0.02 μM (*S*/*N* = 3) was achieved. The further real sample studies demonstrated that the proposed sensor performed excellent in red wine, black tea, and green tea [54].

#### CNT-Based Bioaffinity Electrochemical Biosensors

Bioaffinity sensors, such as DNA biosensors and immunosensors, are based on the recognition and specific binding which happens between two biomolecules. One of the two biomolecules is initially bonded onto the transducer and will be used to capture the target analyte during the detection. Bioaffinity electrochemical sensors collect the measurable electrochemical signal produced by the molecular recognition. CNT-based DNA electrochemical sensors and CNT-based immunosensors will be discussed in the following [47].

##### CNT-Based DNA Electrochemical Sensors

DNA biosensor, based on DNA–DNA hybridization, is of considerable recent interest due to its simplicity, speed, and economical assay for the diagnosis of genetic and infectious diseases and for the detection of genome mutation [55]. When it comes to electrochemical biosensing, a single-stranded DNA (ssDNA) is attached onto an electrode for sensing complementary DNA. An electronic single is directly given by electrochemical reactions caused by the DNA hybridization. However, it is difficult to collect sensitive electrochemical signals for the DNA electrochemical sensor-based electrochemical oxidation of nucleobases (primarily purine) [56]. There are two main reasons: (1) The electrochemical oxidation of purine occurs at high potentials and is characterized by a low electron transfer rate; (2) the peak current is too small to be investigated on classic electrode unless using mercury-based electrode. In order to solve these problems, electroactive indicators such as a cationic metal complex or intercalating organic compound have been used to improve the electrochemical response in DNA electrochemical biosensor. Some other indicator-free designs involve the attachment of the redox group onto the target DNA [57]. With the development of nanomaterials, many researches have demonstrated that the performance of this type of biosensor can benefit from the use of CNTs [58, 59].

DNA oligonucleotides can be immobilized onto the CNT-based electrode through physical absorption [60]. However, covalent attachment plays more important role in CNT-based DNA electrochemical sensors. Cai et al. first reported the use of CNTs to fabricate an electrochemical DNA biosensor for the specific DNA detection. An oligonucleotide probe with amino group at its 5′-phosphate end (NH_2_-ssDNA) was covalently bonded onto the CNTs–COOH-modified glassy carbon electrode (GCE) surface. CNT-modified electrode allows fast electron transfer between electrode and the redox intercalator daunomycin. The DPV measurements were taken from 0.00 to +0.60 V (vs. SCE), and the detection sensitivity achieved 1.0 × 10^−10^ mol L^−1^ for complementary oligonucleotide [61]. In another similar protocol, ssDNA was covalently immobilized to the CNTs-COOH-modified electrode surface and Mn(II) complexes were used as DNA intercalator. ssDNA fragment could be selectively detected with a detection limit of 1.4 × 10^−10^ mol L^−1^ [62]. As we mentioned before, aligned CNTs arrays exhibit quick electron transfer speed, and the use of these nice CNT structures offers promising prospect in fabricating sensitive DNA electrochemical sensors. He et al. demonstrated an effective method to prepare sensitive aligned CNT-based DNA electrochemical sensor. In their protocol, specific DNA sequences were covalently coupled on the tips and sidewalls of plasma-activated aligned CNTs for sensing complementary DNA and/or target DNA chains of specific sequences. The CV results showed that the sensitivity of the DNA electrochemical sensors was 11.36 ng mL^−1^. They concluded that aligned CNTs have implications for advancing the device-level applications of CNT-DNA chips [63]. An ultrasensitive DNA electrochemical sensor based on vertically aligned CNTs embedded in SiO_2_ was reported by Jun et al. Primary amine-terminated oligonucleotides were coupled with terminal –COOH groups on the ends of the CNTs arrays with the assistant of EDC and sulfo-NHS. Ru(bpy)_3_^2+^ mediators were employed to amplify signal for the detection of target DNA. From CV and AC voltammetry (ACV) data, a detection limit lower than a few attomoles of oligonucleotide targets was found [64].

As an important technology in electrochemistry, impedance spectra also have been utilized to observe DNA hybridization without using hybridization marker or intercalator. Xu et al. presented a composite material of polypyrrole (PPy)- and MWNT-based label-free DNA electrochemical sensor by using impedance spectra as detection single. The composite film was electropolymerized onto the electrode in the presence of MWNTs-COOH. Similar to the protocol as mentioned above, ssDNA was covalently coupled with PPy/MWNTs-COOH-modified electrode. A decrease in impedance was observed after the DNA hybridization reaction. It is because that electron transfer resistance of double-stranded DNA is lower than that of ssDNA. In this work, a detection limit of 5 × 10^−12^ mol L^−1^ was achieved for the detection of complementary DNA sequence [65]. Another similar work based on SWNTs was reported by Weber et al., and instead of using conductive polymers, they modified electrode with a mixture of dimethylformamide (DMF) and SWNTs-COOH. This impedance DNA sensor was found to be able to sense complementary target DNA concentration at 1 × 10^−9^ mol L^−1^ [66].

**Scheme 4:**
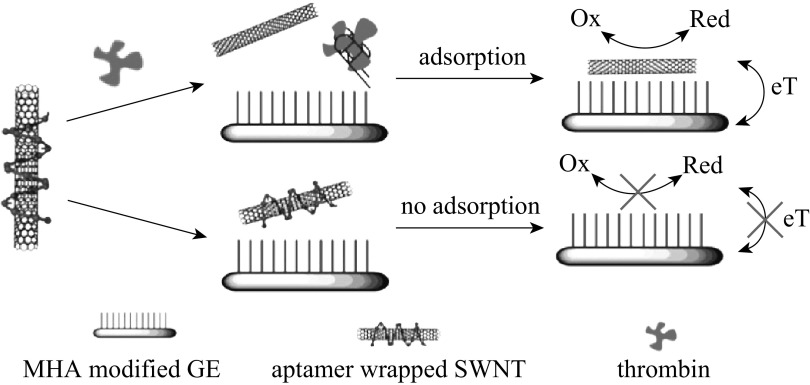
Electrochemical biosensor strategy for thrombin using aptamer-wrapped SWNT as electrochemical labels. Reprinted with permission from Ref. [67]. Copyright (2011) Elsevier

Aptamer-based electrochemical biosensor is another class of DNA sensors. Aptamers are single-stranded DNA/RNA oligonucleotides that bind to their target molecules with high affinity. An aptamer–CNT-based electrochemical biosensor was developed by Guo et al. for detecting thrombin (Scheme 4). An isolating long alkanethiol monolayer 16-mercaptohexadecanoic acid (MHA) was modified on a gold electrode to block the electron transfer between the electrode surface and redox probes. Aptamer was wrapped on the sidewall of CNTs through aromatic interactions. In the presence of thrombin, aptamer was peeled off from the CNTs due to the antibody–antigen interaction. Then the CNTs were free to be assembled on the MHA-modified electrode to mediate efficient electron transfer between the electrode and electroactive species. Additionally, the current increases with the increasing concentration of target protein, and a detection limit of 50 pM thrombin was achieved [67].

##### CNT-Based Electrochemical Immunosensors

**Scheme 5:**
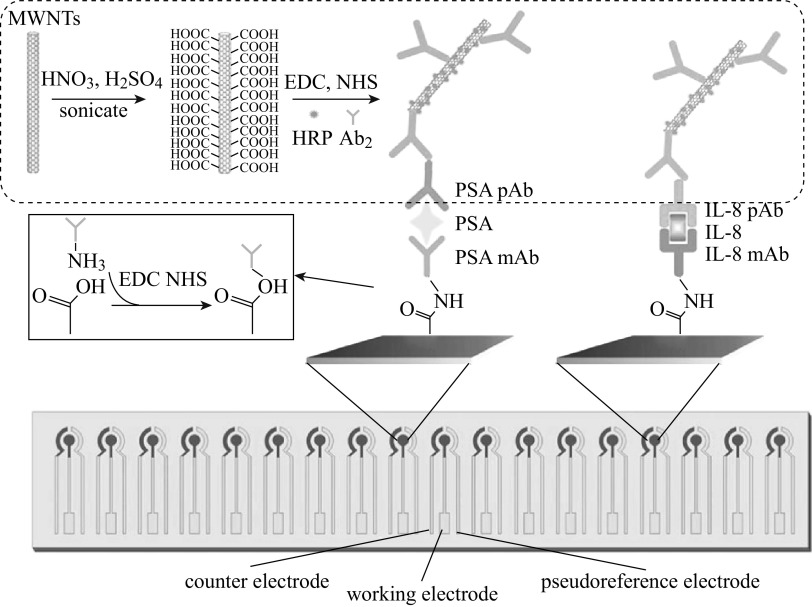
Schematic demonstration for the “sandwich”-type strategy electrochemical immunosensor. Reprinted with permission from Ref. [72]. Copyright (2011) Elsevier

Immunosensors, based on a specific interaction of antibodies with their corresponding antigens, provide a sensitive and selective tool for the detection of many kinds of proteins. Although the antibody–antigen interaction is highly specific, most of them do not yield measurable signals [68]. Electrochemical detection strategies combining with nanomaterials offer opportunities to solve this problem and to achieve highly sensitive protein detection [69]. For nanomaterial-based electrochemical immunosensors, the most common format is sandwich-type assay. In one case, the electrode is coated with nanomaterial first and then modified with capture antibodies. After the attachment of antigens, a secondary antibody conjugate labeled with biomolecules is applied to provide or amply detection signal. In other case, capture antibodies are first coupled on the electrode, followed by the immobilization of antigens. The last step is to introduce a secondary antibody conjugate colabeled with nanomaterial and biomolecules onto the electrode. Aziz et al. described a sensitive electrochemical immunosensor for detecting mouse IgG. An indium-tin-oxide (ITO) electrode comodified with CNTs and poly(ethylene glycol) (PEG)-silane random polymer was applied in this work. Carboxylated CNTs were absorbed onto the electrode with only partial coverage. In order to provide low biofouling properties and minimize the nonspecific binding of proteins, vacant regions of the electrode were covered by a monolayer of PEG-silane copolymer. Avidin was then coupled with the sidewalls of the CNTs to bind biotinylated antimouse IgG. After mouse IgG was attached on the antibody, alkaline phosphatase (ALP)-conjugated antimouse IgG was bound to the mouse IgG. Here, ALP catalyzed the electro-oxidation from *p*-aminophenyl phosphate (APP) to *p*-aminophenol (AP) on the CNTs. The detection limit of 10 pg mL^−1^ was obtained for mouse IgG from CV results, which is much lower compared with the traditional enzyme-linked immunosorbent assays (ELISAs) [70]. As a promising class of polymers in electrochemistry applications, conducting polymer has often been considered attractive for electrochemical biosensors. In the work reported by Gomes-Filho et al., polyethyleneimine (PEI) and COOH–CNTs were coated on a gold electrode. Then anti-cardiac troponin T (cTnT) was bound on the COOH–CNT/PEI electrode. After the immobilization of cTNT, anti-cTnT-HRP was attached on the electrode for the generation of the amperometric signal in H_2_O_2_ solution. As low as 0.02 ng mL^−1^ cTnT was detectable with this sensor [71]. Wan et al. designed an electrochemical immunosensing array platform for simultaneous detection of PSA and IL-8. A screen-printed carbon electrode was applied for the simultaneous detection of cancer biomarkers: prostate-specific antigen (PSA) and interleukin-8 (IL-8). As shown in Scheme 5, the 16-channel disposable SPCE array was firstly activated electrochemically and then modified by mouse monoclonal anti-PSA antibody (PSA mAb) or mouse monoclonal anti-IL-8 antibody (IL-8 mAb). PSA or IL-8 in different concentrations was then immobilized on the sensor platform through antibody–antigen interaction, followed by the attachment of rabbit polyclonal signal anti-PSA antibodies (PSA pAb) or rabbit polyclonal anti-IL-8 antibodies (IL-8 pAb). A universal nanoprobe fabricated by HRP and goat anti-rabbit IgG (Ab_2_)-modified MWNTs was finally coated on the electrode to provide amperometric readout. The authors claimed that they could detect as low as 5 pg mL^−1^ of PSA and 8 pg mL^−1^ of IL-8 with this electrochemical immunosensor [72]. Besides randomly arranged CNTs, vertically aligned CNT array was also employed. Munge et al. presented an electrochemical immunosensor based on vertically aligned CNTs for detecting a cancer biomarker protein matrix metalloproteinase-3 (MMP-3). Similar to the previous protocol, metalloproteinase-3 (MMP-3) antibody (Ab_1_) was first coupled onto the tips of CNTs, followed by the immobilization of antigens MMP-3. A secondary anti-MMP-3 antibody (Ab_2_)–HRP-coated polystyrene beads was applied as amplification element. A ultralow detection limit of 4 pg mL^−1^ in 10 mL serum sample was achieved [73].

For immunosensors using amperometric method, most of them need enzyme or other electroactive labels to provide electrochemical singles. However, the antibody–antigen interaction can be directly detected by impedance spectroscopy without any labels. Hafaiedh et al. reported an electrochemical impedance immunosensors for sensing IgG. The interaction of goat anti-rabbit IgG with different concentration IgG on MWNT-coated electrode was monitored by impedance spectroscopy. The detection limit was found to be 10 pg mL^−1^ [74].

### CNT-Based Field-Effect Transistor (FET) Biosensors

**Scheme 6:**
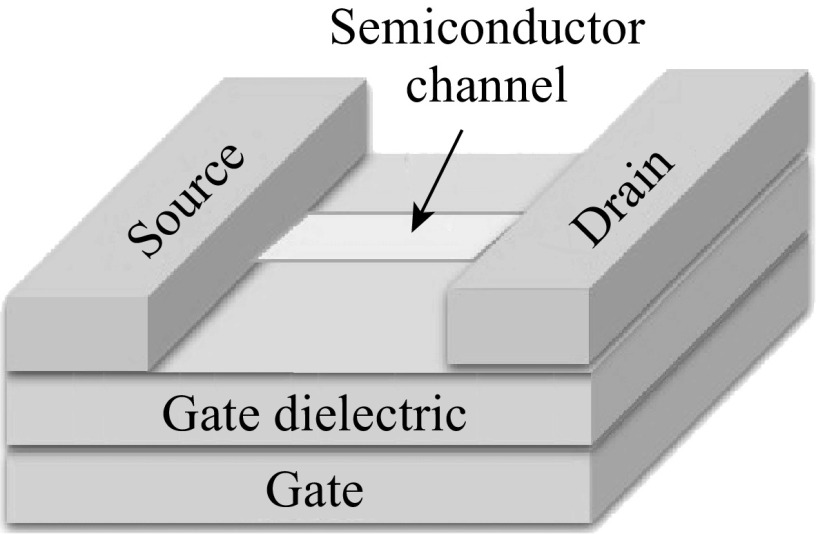
A schematic of field-effect transistor

**Scheme 7:**
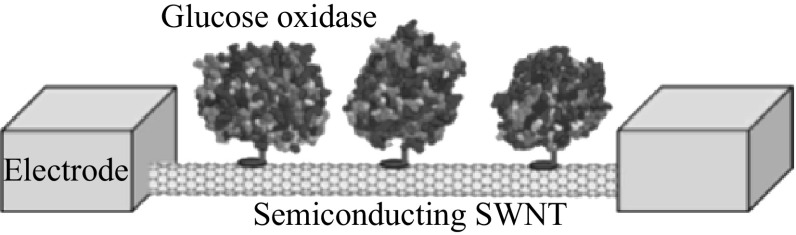
The picture demonstrates two electrodes connected by a semiconducting SWNT with GOx enzymes immobilized on its surface. Reprinted with permission from Ref. [77]. Copyright (2003) American Chemical Society

The field-effect transistor is a semiconductor device, in which the current flows from an electrode (source) on one side to the electrode (drain) on the other side (Scheme 6). The semiconductor channel between the source and drain is controlled by the strength of an electric field produced by a voltage at a third electrode called gate, which is capacitively coupled through a thin dielectric layer [75]. SWNTs can be metallic or semiconducting depending upon the helicity. Semiconducting SWNTs can be used to fabricate FET-based biosensors. The attachment of biomolecules onto the SWNTs and subsequent binding event can change the electrical CNTFET characteristics [76]. A single-molecule-level biosensor based on an individual SWNT was designed by Besteman et al. A linking molecule was modified onto SWNTs through van der Waals coupling with a pyrene group. The other side of the molecule covalently binds to the enzyme glucose oxidase via an amide bond (Scheme 7). A liquid-gate voltage *U*_lg_ was used in the work. They have demonstrated that the designed GOD-coated SWNTs are capable of monitoring enzymatic activity at the single molecule level of an individual SWNT [77].

**Fig. 1:**
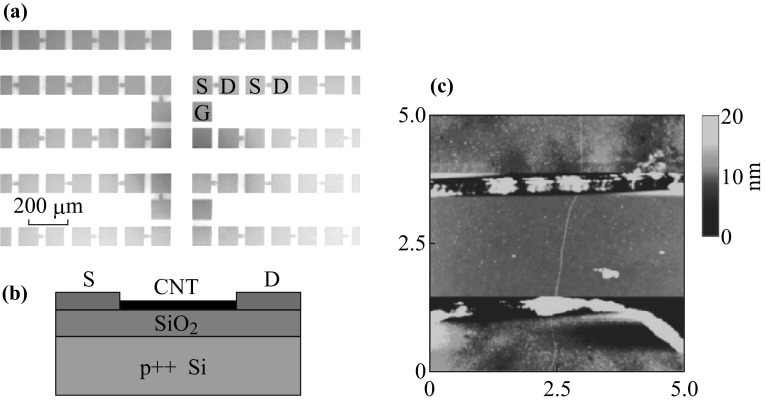
**a** Scanning electron micrograph of the array of CNTFETs. *S* source, *D* drain, *G* contact to back gate. A total of 896 pairs of S/D electrodes are packed in 1 cm^2^. **b** Schematic of device structure. The heavily doped p-type substrate is used as the gate. **c** 5 × 5 µm^2^ AFM picture of a CNTFET device. *Vertical scale*: 20 nm. Reprinted with permission from Ref. [79]. Copyright (2009) American Chemical Society

So et al. reported a SWNT FET biosensor using thrombin aptamers for sensing thrombin. SWNTs were grown on a Si substrate, photolithography and subsequent Ti/Au evaporation, and lift-off techniques were employed to define the source and drain electrodes on the SWNT-FET. Thrombin aptamer was bound onto carbodiimidazole-activated Tween 20-modified SWNTs through covalent bonding. The LOD (lowest detection limit) of the sensor designed in this work is around 10 nM [78]. The electronic detection of DNA hybridization has been carried out by using a carbon nanotube transistor array by Martı´nez et al. Poly(methylmethacrylate_0.6_-co-poly(ethyleneglycol)methacrylate_0.15_-co–*N*-succinimidyl methacrylate_0.25_) was applied to provide connection between CNTs and DNA and simultaneously prevent any other nonspecific adsorption. A large array of back-gated CNTs devices was laid out on a 1-cm^2^ chip. Palladium was used as the contact metal (Fig. 1). They found that statistically significant changes were observed in key transistor parameters after hybridization. It is possible to detect the charge transfer inherent to the hybridization reaction [79]. In the work reported by Oh et al., a CNTs film-based biosensor with a metal semiconductor field-effect transistor structure (CNT-MESFET) was designed for sensing amyloid-β (Aβ) in human serum. A gold top gate was deposited on the middle of the CNTs channel for the immobilization of probe antibodies. In order to increase the density of antibodies immobilized on the sensor surface through their Fc region, Escherichia coli outer membrane (*E. coli* OM) was applied. As an antibody-binding protein, *E. coli* OM has high affinity toward the Fc region of antibodies. Therefore, the free Fc regions of probe antibodies on Au surface lead to an increase in the density of probe antibodies with the proper orientation for binding analytes. Aβ at the level of 1 pg mL^−1^ in human serum could be measured in real time and without labeling using this CNT-MESFET sensor [80].

In order to achieve biocompatible interaction between CNTs and living cells, Sudibya et al. presented a strategy to functionalize SWNTs with bioactive sugar moieties for the detection of dynamic biomolecular release from these cells. *N*-acetyl-d-glucosamine (GlcNAc), d-glucose (Glc), or d-mannose (Man) was anchored onto the nanotube by either a pyrene or a lipid tail. The direct adhesion and growth of PC-12 cells on these glycosylated CNTs networks were examined. They found that GlcNAc-functionalized SWNT nets had better performance than others. Therefore, a GlcNAc-functionalized SWNT-net-based FET biosensor was proposed for the real-time detection of regulated secretion (or exocytosis) of PC12 cells. The influx of Ca^2+^ ion solution in Ca^2+^ ion channels opened by membrane depolarization triggered the actions of fusion of vesicles. Upon the fusion of vesicles, catecholamine molecules inside the vesicle were released onto the narrow interface between the cells and the SWNTs net and then interacted with them by *π–π* stacking. The conductance of nanotube was highly sensitive to the electrochemical perturbations at the surface induced by these interacting molecules. So, the triggered catecholamine molecules released from PC12 cells can be continuously monitored through the changes in current flowed on the surface of nanotubes [81].

### CNT-Based Optical Biosensors

The unique optical properties of CNTs have aroused widespread concerns in development of biosensors during the past few years. Semiconducting SWNTs can act as quenchers for the fluorophores and can display distinctive near-infrared (NIR, wavelength ∼0.8–2 µm) photoluminescence arising from the band-gap fluorescence [82]. Optical biosensors based on these properties have been reported by many research groups. Yang et al. reported a self-assembled quenched complex of fluorescent ssDNA and SWNTs as an efficient molecular beacon (MB) to fluorescently detect single nucleotide variations in DNA. In this design, one end of the ssDNA was labeled with a fluorophore and then assembled onto the surface of SWNTs through *π*-stacking interactions. Here, the SWNTs act as both nanoscaffold and nanoquencher. If the target DNA is not present in the sample, the fluorescently labeled ssDNA-SWNT complexes completely quench the fluorescence. In the presence of the target DNA, the competitive binding of the target and the SWNTs with the ssDNA suppresses the fluorescence quenching, and hence a fluorescence signal was observed. This approach can be extended to design a variety of fluorescent biological probes with detection limits in the nanomolar range [83].

**Fig. 2:**
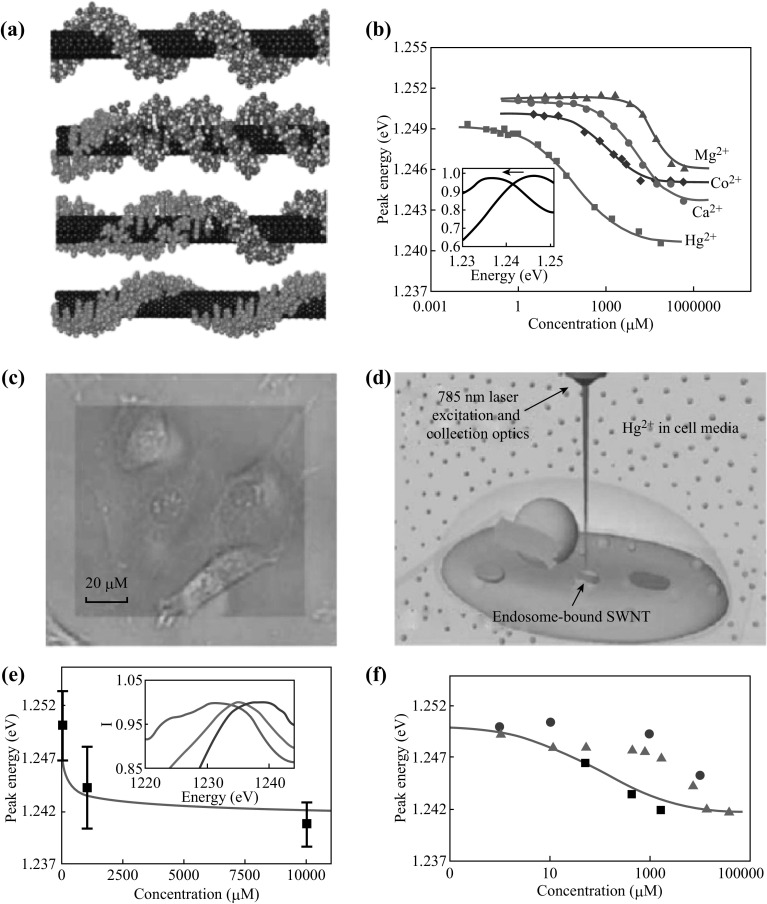
**a** Illustration of DNA undergoing a conformational transition from the B form (*top*) to the Z form (*bottom*) on a carbon nanotube. **b** Concentration-dependent fluorescence response of the DNA-encapsulated (6,5) nanotube to divalent chloride counterions. The *inset* shows the (6,5) fluorescence band at starting (*blue*) and final (*pink*) concentrations of Hg^2+^. **c** Area map of the (6,5) nanotube peak fluorescence intensity of DNA-SWNTs within murine 3T3 fibroblast cells overlaid on an optical micrograph of the same region. **d** Illustration of the experimental method used for ion-binding experiments conducted in mammalian cells. A cell containing endosome-bound DNA-SWNTs undergoes 785-nm excitation through a microscope objective. **e** The (6,5) nanotube fluorescence peak energy of DNA-SWNTs in 3T3 fibroblasts plotted versus Hg^2+^ concentration in the cell medium. The fluorescence energy of a population of 8–10 cells was averaged for each data point. *Error bars* indicate 1SD. The *red line* shows the model curve from original Hg^2+^ binding experiment conducted in Tris buffer. The *inset* shows individual spectra at each concentration. **f** The (6, 5) nanotube fluorescence energy of DNA-SWNTs in the following highly absorptive and scattering media: whole rooster blood (*green triangles*), black dye solution (*black squares*), and chicken tissue (*blue circles*) plotted on a model curve (*red*) from Hg^2+^ addition to SWNTs in buffer. The Δ*E* of all blood and tissue data points was corrected for an initial redshift due to the cellular environment. Reprinted with permission from Ref. [85]. Copyright (2006) American Association for the Advancement of Science. (Color figure online)

As we mentioned before, semiconducting SWNTs exhibit photoluminescence in the NIR due to the small band gaps. As a NIR fluorophores, semiconducting SWNTs can be used to develop nanoscale biosensors that could detect and image sensitive molecular in confined environment such as inside cells [84]. The band-gap energy of SWNTs is sensitive to the dielectric environment, and Heller et al. designed an optical biosensor for the detection of DNA conformational polymorphism on SWNTs. In their work, a complex of DNA-SWNT was synthesized by the noncovalent bond between the nanotube sidewall and a 30 base pair single-stranded DNA (ssDNA) oligonucleotide with a repeating G-T sequence. This ssDNA can form hydrogen bond with each other to form dsDNA. The adsorption of divalent cations onto the negatively charged DNA backbone can induce a transition from the native, right-handed B form to the left-handed Z form (Fig. 2a). This B–Z form change results in a change of the dielectric environment of the SWNTs with an energy shift in the SWNTs emission. The order of the sensitivity of the relative ions is: Hg^2+^ > Co^2+^ > Ca^2+^ > Mg^2+^ (Fig. 2b). They also localized DNA-SWNTs within murine 3T3 fibroblasts and added various concentrations of HgCl_2_ (Fig. 2c). It can be observed from the inset of Fig. 2e, and the SWNTs emission redshifts with increasing Hg^2+^ concentration. After correcting the initial shift caused by the new environment, the peak energy of DNA-SWNTs in 3T3 fibroblasts in the cell medium fits the model curve from original Hg^2+^ binding experiment conducted in Tris buffer. From Fig. 2f, Hg^2+^ was still detectable in the media that possess a strong ionic background. It means that this optical biosensor can detect the B–Z change in whole blood, tissue, as well as living mammalian cells [85].

SWNTs also can be utilized as NIR fluorescent tags for selective probing and imaging cells. In the work reported by Welsher et al., polyethyleneglycol (PEG)-modified SWNTs are conjugated to Rituxan antibodies to selectively recognize CD20 cell surface receptor on B cells with little nonspecific binding to negative T cells and Herceptin antibodies to recognize HER2/neu-positive breast cancer cells. The selective SWNT antibody binding to cells was imaged by detecting intrinsic NIR photoluminescence of the nanotubes [86].

**Fig. 3:**
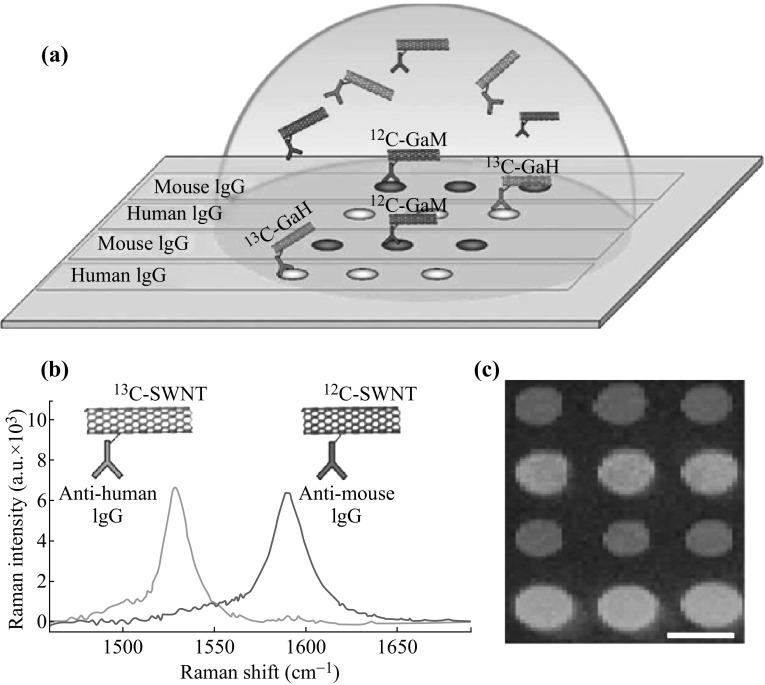
Multicolor SWNT Raman labels for multiplexed protein detection. **a** Two-layer, direct, microarray-format protein detection with distinct Raman labels based upon pure ^12^C and ^13^C SWNT tags. ^12^C and ^13^C SWNTs were conjugated to GaM and GaH-IgGs, respectively, providing specific binding to complimentary IgGs of mouse or human origin, even during mixed incubation with analyte (as shown). **b** G-mode Raman scattering spectra of ^12^C (*red*) and ^13^C (*green*) SWNT Raman tags are easily resolvable, have nearly identical scattering intensities, and are excited simultaneously with a 785-nm laser. This allows rapid, multiplexed protein detection. **c** Raman scattering map of integrated ^12^C (*red*) and ^13^C (*green*) SWNT G-mode scattering above baseline, demonstrating easily resolved, multiplexed IgG detection based upon multicolor SWNT Raman labels. *Scale bar*, 500 µm. Reprinted with permission from Ref. [87]. Copyright (2008) Nature Publishing Group. (Color figure online)

Another important optical property for SWNTs is that they exhibit strong Raman scattering. Chen et al. used antibody-modified SWNTs as multicolor Raman labels for highly sensitive, multiplexed protein detection in an arrayed format. As shown in Fig. 3, human IgG and mouse IgG were immobilized in two sets, each with three 400-nm-diameter spots on gold-coated glass slides. ^12^C and ^13^C isotopic SWNTs were synthesized and conjugated to goat anti-mouse immunoglobulin G (GaM-IgG) and goat anti-human immunoglobulin G (GaH-IgG), respectively. The mixture of these two bioconjugates was incubated on the sensing platform, leading to specific binding to IgG of mouse or human origin with high selectivity. From the G-mode Raman scattering spectra, a redshift in the G-peak positions was observed for ^13^C bioconjugate due to the isotope effect, which allows the simultaneous detection of two types of IgGs. They found that the use of multicolor SWNTs Raman labels enabled the simultaneous detection of multiple proteins with a high sensitivity of 1 fM on a multiplexed sensing platform [87]. Gold-functionalized vertically aligned carbon nanotube forests (VACNTs) as low-cost straightforward surface-enhanced Raman scattering (SERS) nanoplatforms were reported by Goldberg-Oppenheimer et al. They found that SERS enhancements of CNTs forest substrates highly depended on their diameter and density. The performance of the VACNT-based SERS substrates can be turned by altering above structural parameters. The finally proposed micropatterned gold-VACNTAs platforms were found to give multiplexed SERS detection [88].

Electrochemiluminescent (ECL) is a luminescence process that is produced by electrochemical reactions in solution. A CNTs microwell array-based ECL biosensor for detection of cancer biomarkers was developed by Sardesai et al. SWNTs forests were coated onto each of microwells on a pyrolytic graphite (PG) chip. Cancer biomarker prostate-specific antigen capture antibodies (PSA-Ab_1_) and interleukin-6 capture antibodies (IL-6-Ab_1_) were covalently coupled onto the SWNTs forest for capturing protein analytes. Silica nanoparticles containing [Ru(bpy)_3_]^2+^ and secondary antibodies (RuBPY-silica-Ab_2_) were used as the signal-amplified element. [Ru(bpy)_3_]^2+^ labels produce ECL in a multistep electrocatalytic redox reactions with a suitable sacrificial reductant such as tripropylamine (TprA) to yield photoexcited [Ru(bpy)_3_^2+^]* that emits light at 610 nm. ECL light intensity was integrated by the CCD camera. The detection limit for PSA was 1 pg mL^−1^ and for IL-6 was 0.25 pg mL^−1^ in serum. These SWNT forest arrays can be used to interfacing with microfluidic for simultaneous detection of different types of proteins [89].

## Graphene-Based Biosensors

Graphene, a 2D carbon material with one-atom thickness, has become one of the hottest research topics in the field of biosensors. Similar to CNTs, *sp* ^2^-bonded carbon atoms in graphene are closely packed in a honeycomb lattice structure. Owing to their unusual structure, graphene and its derivatives exhibit several extraordinary properties including high thermal conductivity, tunable optical property, high planar surface, superior elasticity, and mechanical strength [90]. In addition, many research results have revealed that graphene and its derivatives possess remarkable electronic properties, such as a high quantum Hall effect at room temperature [91], an ambipolar electric field effect along with ballistic conduction of charge carriers [92], electron–hole symmetry, and internal degrees of freedom [93]. These notable properties make graphene an attractive candidate for the development of the new generation of biosensors with outstanding performance [94, 95]. Currently, there are several physical and chemical methods for producing graphene and graphene-related materials, such as mechanical exfoliation of bulk graphite, chemical vapor deposition (CVD) of hydrocarbons on metal substrates, and chemical or thermal exfoliation of graphite oxide to graphene oxide sheet [94]. Among them, chemical or thermal exfoliation has attracted much attention because of easy operation and high yields of bulk-quantity graphene and graphene oxide. Based on different reduction approaches, thermally reduced graphene oxide (TRGO), chemically reduced graphene oxide (CRGO), and electrochemically reduced graphene oxide (ERGO) can be produced [36]. Although made with different methods, all graphene products made by these methods contain large amounts of defects and oxygen-bearing groups. The presence of these defects can be beneficial to the further functionalization of chemical or biomolecules [96].

To employ CNTs or graphene in biosensors, chemical functionalization has to be made to enable a biocompatible surface, which is able to be further conjugated with other molecules. The sensing performance is highly dependent on the functionalization process. As we mentioned, CNTs can be chemically functionalized by both covalent and noncovalent methods. Chemical functionalization is also of great importance to graphene to render high water solubility and biocompatibility; for example, diazonium chemistry has been used to graft various functional groups onto graphene surface [97], and many aromatic molecules can be physically absorbed onto graphene surface through *π–π* stacking [98]. After the pre-functionalization, graphene is much easier to be modified by other chemical and biological compounds for further sensing applications [99, 100]. Graphene-based electrochemical biosensors, field-effect transistors, and optical biosensors have greatly stimulated research interests in the past few years and will be reviewed in the following part.

### Graphene-Based Electrochemical Biosensors

It has been reported that heterogeneous electron transfer of *sp* ^2^ carbons occurs at the edges and defects not at the basal plan of graphene sheets [101, 102]. Oxygen containing groups on GO surface and the super high surface area provided by its 2D structure can enhance the electron transfer rate and are advantageous for improving the sensitivity of electrochemical biosensors [96, 103]. Graphene-based electrochemical biosensors thus are of great interest to researchers in recent years.

#### Graphene-Based Enzymatic Electrochemical Biosensors

As we discussed in the above, CNTs have exhibited excellent performance in enhancing the direct electron transfer between various enzymes and electrodes. The ongoing research shows that graphene-related materials can also promote the electron transfer between enzymes and electrode due to their extraordinary electron transport property and high surface area [94, 104]. The common approaches for immobilization of enzyme onto graphene materials are similar to that onto CNTs surface. Enzymes can be noncovalently or covalently attached onto the electrode modified with graphene materials. In the work reported by Shan et al., GO was exfoliated from graphite. PVP-protected GO was then prepared and found to have good electrochemical reduction toward H_2_O_2_ and O_2_. A carboxyl-terminated ionic liquid polyethylenimine-functionalized ionic liquid (PFIL) was applied to be covalently attached onto PVP-protected GO for the immobilization of glucose oxidase. Counter anions were allowed to be exchanged with negatively charged GOx. The amperometric response of graphene-GOx-PFIL biosensor is linear against the concentrations of glucose ranging from 2 to 14 mM [105]. Liu et al. reported a glucose biosensor through the covalent attachment between carboxyl acid groups on GO sheets and the amine groups of GOx. The amperometric responses at the covalently linked GOx-GO enzyme electrode were examined, and the sensitivity was found to be 8.045 mA cm^−2^ M^−1^ [106]. It has been reported that doping with heteroatoms such as nitrogen is an excellent method to provide pathways for efficient electron transfer processes. Prathish et al. have developed and compared various modified electrodes with basic or acidic functionalized graphene and nitrogen-doped graphene (NG) as well as with composite materials of NG, conducting polymer poly(3,4-ethylenedioxythiophene) (PEDOT) and the redox polymer poly(PNR). Among these electrodes, the N-graphene-modified GCE has shown higher sensitivities than the other electrodes for the detection of cofactors present in oxidase and dehydrogenase-based enzymes: nicotinamide adenine dinucleotide (NADH) and flavin adenine dinucleotide (FAD), due to its n-type semiconductor like properties [107]. The layer-by-layer technique has been used by Barsan et al. for the construction of a glucose biosensor based on multilayer films containing chitosan biopolymer, poly(styrene sulfonate) (PSS), GOx, and nitrogen-doped graphene. GOx and NG were dispersed in the biocompatible positively charged polymer chitosan (chi + (NG + GOx)) and coated onto a gold electrode surface. Then the negatively charged PSS, PSS−, was assembled onto the electrode. This process was repeated for further bilayers. They found that the presence of N-doped graphene decreases the charge transfer resistance of the assembly and provides a film matrix that presents significant charge separation. The biosensor operates at a low potential of −0.2 V versus Ag/AgCl, exhibiting a high sensitivity of 10.5 μA (cm^−2^ mM^−1^) and a detection limit of 64 μM [108]. Zhang et al. compared the loading amount of enzymes on GO and chemically reduced graphene oxide (CRGO). They found that the enzyme can be adsorbed onto CRGO directly with a tenfold higher loading than that on GO. The immobilizations of HRP and oxalate oxidase (O_*x*_O_*x*_) on CRGO were reported. The maximum enzyme loadings were found to reach 1.3 and 12 mg mg^−1^ for HRP and O_*x*_O_*x*_, respectively. The results indicate that CRGO is a potential promising substrate for efficient enzyme immobilization [109].

Besides the graphene-based enzymatic biosensors, the research on graphene-based nonenzymatic biosensors has been carried out worldwide. Wang et al. reported a nonenzymatic glucose biosensor based on Pd nanoparticle-modified graphene oxide (Pd NPs/GO). A linear range of 0.2–10 mM with high selectively was achieved [110]. A novel structure of Au nanoparticle-decorated three-dimensional (3D) porous reduced graphene oxide-single-walled carbon nanotube (rGO-SWCNT-Au) nanocomposites was proposed by Luo et al. for sensing glucose. Their method could achieve the simultaneous reduction of GO and HAuCl_4._ The prepared rGO-SWCNT-Au nanocomposites were utilized to monitor glucose and revealed a wide linear range and a low detection limit [111]. Bimetallic nanoparticles also have been used for enzymeless biosensors, and Luo et al. developed a PdCu nanoparticle-decorated 3D graphene hydrogel-based biosensor for the detection of glucose. The synthesized nanohybrids showed a high sensitivity of 48 μA (mg mM)^−1^ with a wide linear range up to 18 nM toward glucose oxidation [112].

**Scheme 8:**
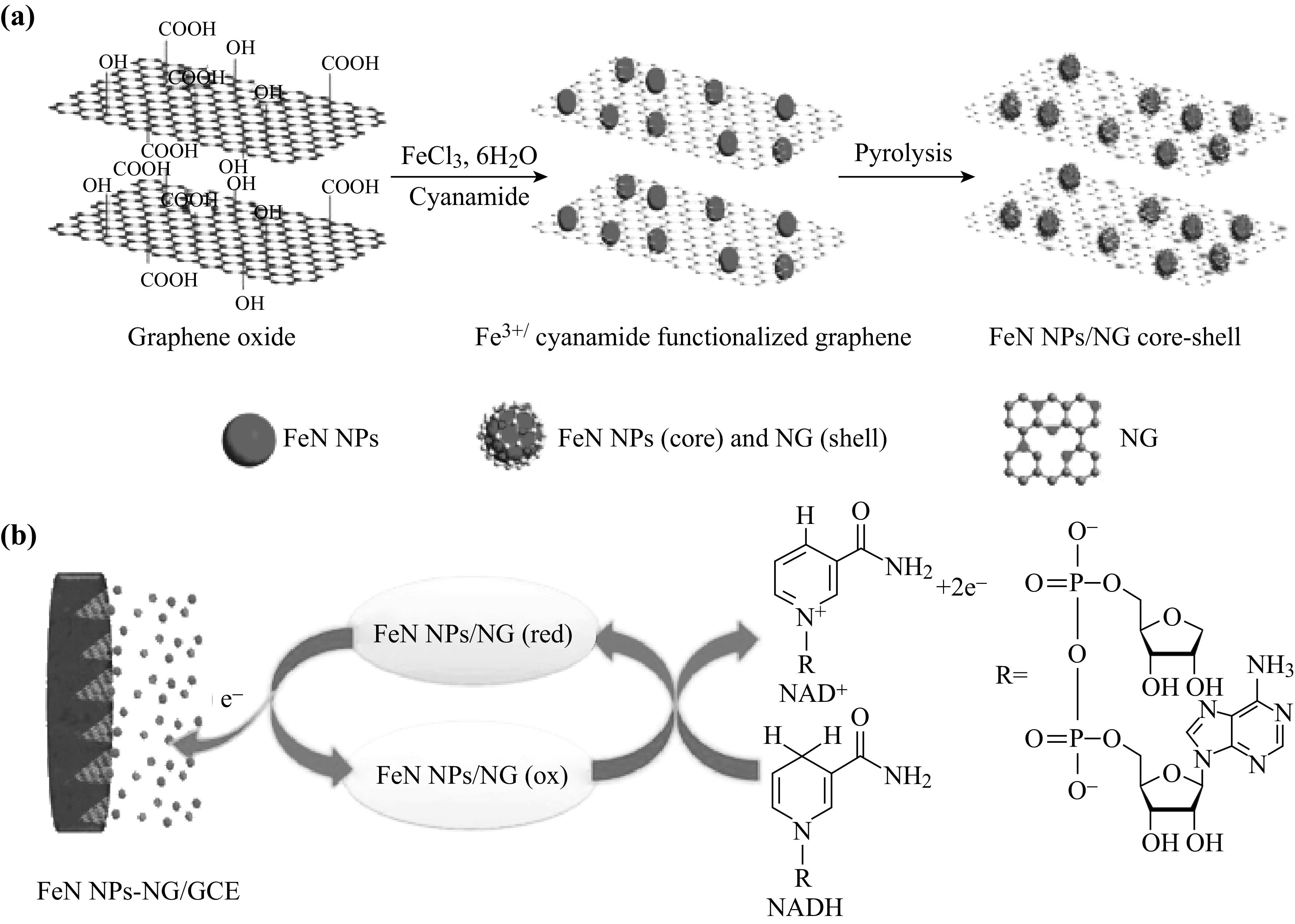
**a** Fabrication of the hierarchical FeN NPs/NG core–shell. **b** The proposed NADH measurement mechanism on a FeN NPs/NG core–shell hybrid-based electrode. Reprinted with permission from Ref. [113]. Copyright (2016) Elsevier

Balamurugan et al. recently reported a novel strategy for the synthesis of an iron nitride nanoparticles/nitrogen-doped graphene (FeN NPs/NG) core–shell hybrid through single-step synthesis method as shown in Scheme 8. The produced core–shell hierarchical nanostructure offers excellent conductive and large surface area, resulting in improved electrocatalytic activity toward the direct electrochemical oxidation of NADH. The as-prepared enzymeless sensor displays a high sensitivity of 0.028 µA (µM^−1^ cm^−2^), a wide linear range from 0.4 to 718 µM, and a low detection limit of 25 mM with a fast response time of less than 3 s. The further study in human blood serum was performed, and the proposed FeN NPs/NG core–shell hierarchical nanostructure was found to be a promising candidate for electrochemical biosensor with high sensitivity, excellent selectivity, and good stability [113]. A straightforward measurement system for simultaneous determination of melatonin (MT) and serotonin (5-HT) based on carbon screen-printed electrodes (CSPEs) has been developed by Gomez et al. Several carbon nanomaterials (SWNTs, MWNTs, graphene oxide nanoribbons, and reduced graphene oxide nanoribbons) were drop casted on CSPEs from ammonia solution for the examination. The electrode active area of carbon nanomaterial-modified electrodes is stated in the following increasing order: MWCNTs < SWCNTs < graphene. The highest sensitivity for the simultaneous determination of 5-HT and MT was obtained from graphene oxide nanoribbon-based electrode at the potentials of 0.05 V for 5-HT and 0.39 V for MT (vs. Ag) in DPV measurements, respectively. They found that carbon nanomaterial-modified electrode can be used as an excellent platform for simultaneous sensing in even small sample volumes [114].

#### Graphene-Based Bioaffinity Electrochemical Biosensors

##### Graphene-Based DNA Electrochemical Sensors

Graphene’s large 2D aromatic surface is an ideal substrate for adsorption of biomolecules as well as a burgeoning support to disperse and stabilize metal, metal oxide, and semiconductor nanomaterials [90]. Similar to CNT-based DNA biosensors, in order to collect sensitive current signal, electroactive indicators often used to provide and improve the electrochemical response. Zhu et al. reported a DNA electrochemical biosensor based on thionine–graphene nanocomposite. Thionine–graphene was covalently modified onto a gold electrode surface with the use of l-cysteine (Cys) and cross-linker glutaraldehyde (GA). Then, the probe ssDNA was covalently grafted onto the electrode with the presence of GA. The hybridization procedure was monitored by DVP analysis using an electroactive intercalator daunomycin on the electrode. The complementary oligonucleotide could be quantified in a wide range of 1.0 × 10^−12^ to 1.0 × 10^−7^ M with a good linearity and a low detection limit of 1.26 × 10^−13^ M [115]. Using graphene as signal amplification element is also a very common strategy in graphene-based DNA biosensors. Wang et al. presented a DNA biosensor based on using graphene-supported ferric porphyrin as a peroxidase mimic. A biotinylated molecular beacon (MB) was coated on a gold nanoparticles single-walled carbon nanohorn (AuNPs–SWCNH) composite-modified glassy carbon electrode. In the presence of target DNA, the MB probe “opened” and the biotin group moved away from the electrode surface. Then, a porphyrin-based HRP mimic as a trace label was introduced through the specific interaction between streptavidin on the trace label and biotin group on the electrode. In their work, an iron porphyrins iron (III) meso-tetrakis (*N*-methylpyridinium-4-yl) porphyrin (FeTMPyP) was used to mimic the biological function of HRP. Due to the 2D structure of graphene, FeTMPyP could be attached on the both sides of graphene and led to a high loading amount. They found that this method showed a detection limit down to attomolar levels, much lower than that obtained with a HRP-based trace label [116].

**Fig. 4:**
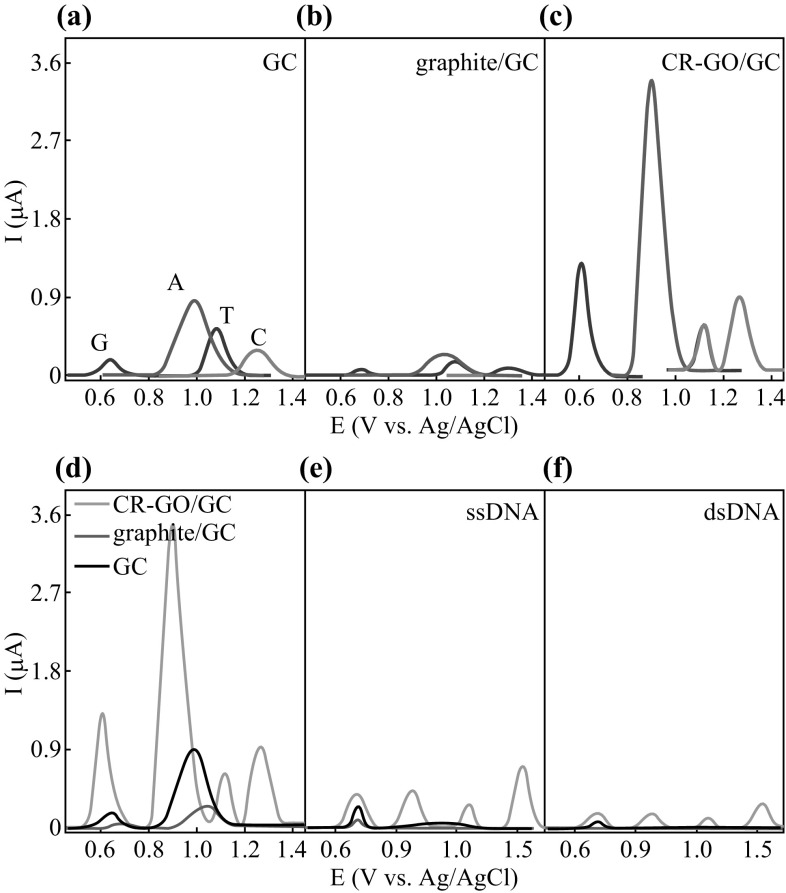
**a** DPVs at the GC electrode for G (*blue*), A (*orange*), T (*violet*), and C (*magenta*), respectively. **b** DPVs at the graphite/GC electrode for G (*blue*), A (*orange*), T (*violet*), and C (*magenta*), respectively. **c** DPVs at the CR-GO/GC electrode for G (*blue*), A (*orange*), T(*violet*), and C (*magenta*), respectively. **d** DPVs for a mixture of G, A, T, and C at CR-GO/GC (*green*), graphite/GC (*red*), and GC electrodes (*black*). **e** DPVs for ssDNA at CR-GO/GC (*green*), graphite/GC (*red*), and GC electrodes (*black*). **f** DPVs for dsDNA at CR-GO/GC (*green*), graphite/GC (*red*), and GC electrodes (*black*). Concentrations for different species **a**–**f**: G, A, T, C, ssDNA or dsDNA: 10 µg mL^−1^. Electrolyte: 0.1 M pH 7.0 PBS. Reprinted with permission from Ref. [117]. Copyright (2009) American Chemical Society. (Color figure online)

Instead of using electroactive indicators or enzyme labels, DNA hybridization can be directly detected through the oxidative signals of DNA bases. As we know, only the adenine (A) and guanine (G) bases give detectable useful signals on traditional carbon electrodes [96]. In the work reported by Zhou et al., chemically reduced graphene oxide (CRGO)-modified electrode gives analytical useful signals for all four free bases DNA (guanine (G), adenine (A), thymine (T), and cytosine (C)) with higher sensitivity in contrast to the bare glassy carbon electrode and graphite-modified GC electrode (Fig. 4a–d). This is due to the high density of defective and active sites on CRGO which would be beneficial for accelerating electron transfer between the electrode and the species in electrolyte. This feature also facilitates the label-free direct detection of bases in ssDNA and dsDNA based on the oxidation of four bases (Fig. 4e–f). Moreover, a wide type oligonucleotide and its single base mismatch can be discriminated through the changes of oxidation peaks of four DNA bases [117].

Graphene-based DNA impedimetric sensor has also attracted a significant amount of attention due to their advantage of being indicator free. Bonanni et al. have demonstrated a label-free electrochemical impedance DNA biosensor for the detection of DNA hybridization and polymorphism. They also compared the performance of sensors with different graphene layers. Different graphene platforms were modified with hairpin-DNA (hpDNA) probes for the sensitive detection of a single nucleotide polymorphism (SNP) correlated with the development of Alzheimer’s disease. The negatively charged hpDNA probes were immobilized on the graphene and caused an increase in electron transfer resistance. After hybridization with wide type target, the electron transfer resistance decreased due to the partial release of the hpDNA probes from the electrode surface. The less resistance decrease was observed for the hybridization with mutant, and no decrease was observed with the noncomplementary sequence. They also found that sensors with four layers of graphene provided the best sensitivity and were employed for the detection of SNPs. A higher sensitivity was found for as-prepared impedance DNA biosensor compared to that obtained with similar platform using fluorescence methods [118]. In the work reported by Zhang et al., DNA probe was grafted onto the tryptamine (TRA)-functionalized reduced graphene oxide (TRA–RGO) through the cross-linker GA. DNA hybridization reaction is monitored by EIS. The as-prepared electrochemical impedance DNA biosensor shows excellent reproducibility and a detection limit of 5.2 × 10^−13^ M [119].

##### Graphene-Based Electrochemical Immunosensors

As one of the most promising nanomaterials, graphene has been widely used in electrochemical immunosensors. An electrochemical biosensing platform based on graphene for analysis of vascular endothelial growth factor receptor 2 (VEGFR 2) was proposed by Wei et al. In their work, a sandwich-type assay format based on thionine-functionalized chitosan-reduced graphene oxide (CHI-RGO) electrode was developed to detect VEGFR 2 in cell lysates directly. Capture antibodies were grafted onto RGO through the cross-linker GA for the adsorption of VEGFR 2, followed by the immobilization of biotinylated detection antibody. A bioconjugate of antibody and streptavidin-HRP was then used as enzyme labels to provide current signals. The detection limit for VEGFR 2 was found to be 0.28 pM [120]. Liu et al. presented a graphene-assisted dual amplification strategy for the fabrication of sensitive amperometric immunosensor for detection of human IgG (HIgG). In their protocol, a composite of poly(diallyldimethylammonium chloride)-functionalized graphene nanosheets (PDDA-G) and gold nanoparticles (AuNPs/PDDA-G) was firstly coated on the electrode, followed by the immobilization of probe antibodies. The target HIgG antigen was then assembled onto the electrode through antibody–antigen interactions. In order to achieve a high sensitivity for the detection of HIgG, the authors applied HRP–detection antibody/AuNPs/PDDA–EGO bioconjugation to fabricate a sandwich-type assay format. The proposed methods showed excellent analytical performance for the detection of HIgG ranging from 0.1 to 200 ng mL^−1^ with a detection limit of 0.05 ng mL^−1^ [121].

The label-free graphene-based impedimetric immunosensors have been developed by many researchers. An electrochemical impedance-based biosensor for determination of bovine interleukin-4 (bov-IL-4) was proposed by Chen et al. Reduced graphene oxide (RGO)/chitosan composites were modified on glassy carbon electrode for the attachment of monoclonal bov-IL-4 antibody. The resultant electrochemical impedance is linearly proportional to its logarithmic concentration in the range from 0.1 to 50 ng mL^−1^, and the detection limit is 80 pg mL^−1^ [122]. Mishra et al. reported a novel electrochemical impedimetric immunosensors for the quantitative detection of human cardiac myoglobin (Ag-cMb). In their protocol, 3-mercaptopropionic acid (MPA)-capped ZnS nanocrystals (ZnS(MPA)) were anchored on RGO sheets through a cross-linker and deposited onto silane-modified indium-tin-oxide (ITO) glass plate. ZnS nanocrystals allow greater affinity with the target biomolecules and provide a favorable environment to retain the activity of the antibodies. The protein antibody, Ab-cMb, was then covalently coupled onto ZnS–RGO nanocomposite to capture antigen Ag-cMb. They found that the ZnO–RGO hybrid-modified electrode exhibited a higher sensitivity of about 2.5-fold higher than that of the bare RGO-modified electrode. The proposed immunosensors exhibited a linear electrochemical impedance response to Ag-cMb from 10 ng mL^−1^ to 1 mg mL^−1^ with a sensitivity of 177.56 Ω cm^2^ per decade [123].

**Fig. 5:**
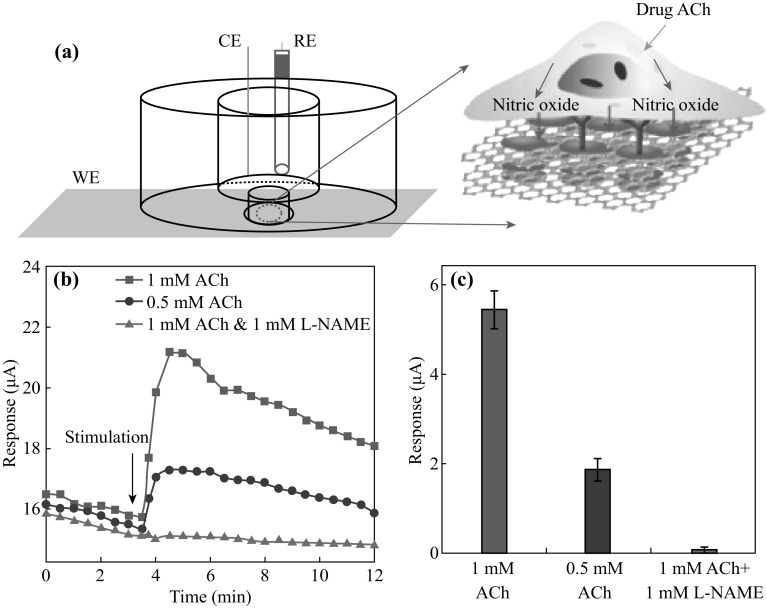
**a** The setup for live-cell assay. **b** Real-time monitoring of nitric oxide molecule released from the attached cells on RGD-peptide covalently bonded graphene biofilm in cell culture medium. The drug was added at the time indicated by the *arrow*. **c** The current responses of the cultured cells on RGD-peptide covalently bonded graphene biofilm toward different drugs. Acetylcholine (Ach) is a model drug to stimulate cell nitric oxide release, and NG-nitro-L-arginine methyl ester (L-NAME) is a specific nitric oxide inhibitor. Reprinted with permission from Ref. [124]. Copyright (2012) American Chemical Society

Real-time detection of biomolecules released from living cells is significant in the development of applications in clinical diagnosis and drug discovery. Guo et al. reported a biomimetic living cell sensors based on RGD-peptide-functionalized graphene for real-time detection of the biological signaling molecule, nitric oxide. As shown in Fig. 5, RGD peptide was covalently bound onto pyrenebutyric acid-functionalized chemical reduced graphene oxide surface to significantly boost cell adhesion and growth for real-time electrochemical detection of nitric oxide molecule released from attached human cells under drug stimulations. They found that the RGD-peptide modification makes the film biomimetic for human cell attachment and growth. The biofilm-cultured human endothelial cells were used to demonstrate the real-time detection of nitric oxide molecule released from attached cells. Acetylcholine (Ach) and NG-nitro-L-arginine methyl ester (L-NAME) were used as a model drug to stimulate cells to release and inhibit the release of nitric oxide. The nitric oxide concentration is around 350 and 145 nM for 1 and 0.5 mM Ach stimulation, respectively. Since the cell could continue to grow on the biofilm, it was claimed that the method may simultaneously monitor the time-dependent cell density while detecting nitric oxide [124].

### Graphene-Based Field-Effect Transistor (FET) Biosensors

**Scheme 9:**
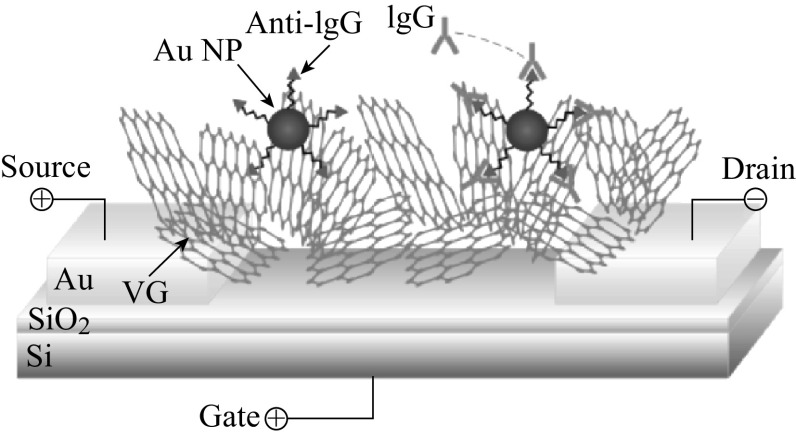
The VG FET sensor is made by direct growth of VG between the drain and the source electrodes. Probe antibody is labeled to the VG surface through Au NPs. Reprinted with permission from Ref. [127]. Copyright (2013) Nature Publishing Group

Recently, much attention has been given to graphene-based FET biosensor due to its high sensitivity to electrical perturbations and high carrier mobility. Ohno et al. reported a label-free immunosensor based on an aptamer-modified graphene field-effect transistor (G-FET) for the electrical detection of IgE protein. The immobilization of IgE aptamers was confirmed by AFM observations. The height of the aptamers is about 3 nm. The aptamer-modified G-FET specifically detected IgE protein [125]. Mao et al. fabricated a highly sensitive FET biosensor using thermally reduced graphene oxide (TRGO) sheets functionalized with Au NPs. Probe antibody anti-IgG was labeled on the surface of TRGO sheets through Au NPs for electrical detection of IgG. The protein binding was monitored by FET and direct current (dc) measurements. They found that the sensor response was more significant with larger TRGO base resistance and higher antibody areal density. A lower detection limit of 0.2 ng mL^−1^ was achieved [126]. In another work reported by the same group, instead of using randomly arranged graphene, vertically oriented graphene (VG) was applied to fabricate a sensitive and selective FET biosensor. VG sheets were directly grown on the gold electrode and then modified with Au NP-antibody conjugates (Scheme 9). This novel biosensor shows high sensitivity (down to ~2 ng mL^−1^ or 13 pM) and selectivity toward specific proteins [127]. You et al. utilized silk protein as both device substrate and enzyme immobilization materials to develop silk fibroin-encapsulated graphene FET enzymatic biosensors for sensing glucose. The silk fibroin can maintain enzyme activity at room temperature for a long time. The detection limit of the fabricated biosensors was approximately 0.1 mM [128].

**Scheme 10:**
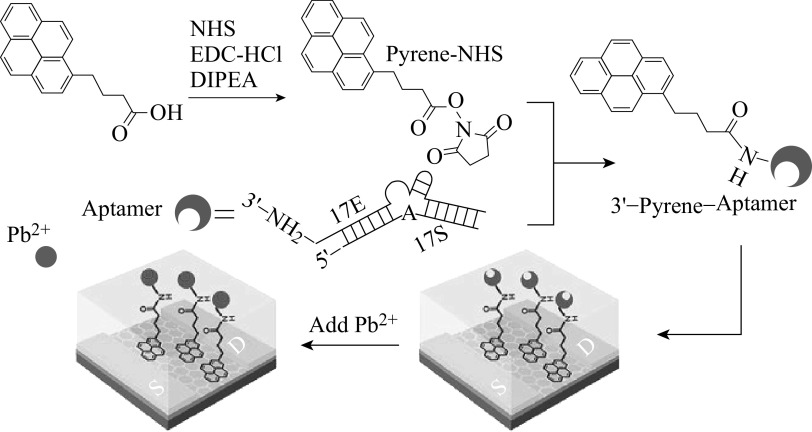
Illustrative scheme of the preparation of graphene field-effect transistor. Reprinted with permission from Ref. [129]. Copyright (2016) Nature Publishing Group

Wang et al. proposed a label-free and portable aptasensor based on graphene FET for the detection of child blood lead. Briefly, 8–17 DNAzymes were functionalized on the graphene surface for the capture of Pb^2+^ (Scheme 10). A low detection limit of 37.5 ng L^−1^ (three orders lower than the safe blood lead level: 100 μg L^−1^) was obtained in standard solutions with different Pb^2+^ concentrations. The proposed aptasensor was further used to detect Pb^2+^ in real blood sample from children. Their results suggested that such graphene FET aptasensors have great potential for future applications on monitoring of heavy metal ions for health care and diagnosis [129]. The use of FET sensor for the detection of cell signals has been demonstrated by Hess et al. Graphene-based solution-gated field-effect transistors (G-SGFETs) for the detection of the electrical activity of electrogenic cells were proposed. In their protocol, cardiomyocyte-like HL-1 cells were cultured on graphene films and exhibited a healthy growth. The action potentials of these cells were measured after in an electrolyte by the S-SGFETs with the cells. The low noise of G-SGFETs and the large transconductive sensitivity of this device confirmed the advantage by combining SGFETs with graphene [130].

### Graphene-Based Optical Biosensors

**Scheme 11:**
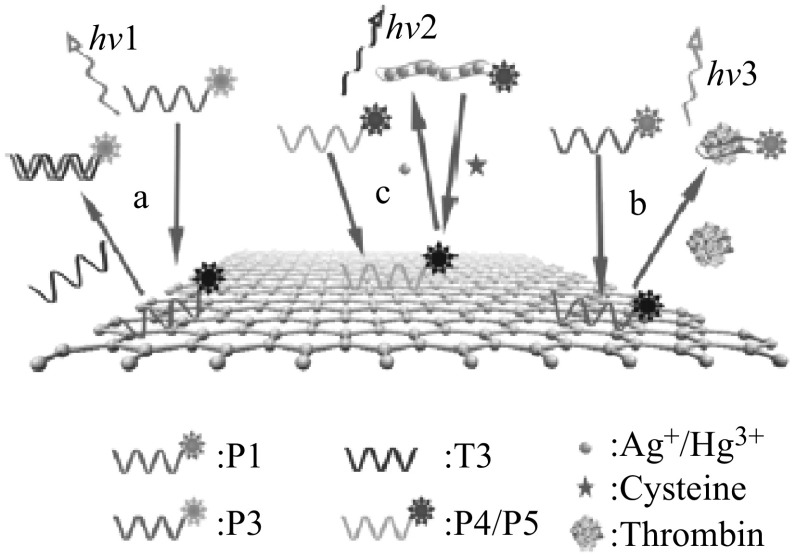
Schematic representation of the ssDNA-GO architecture platform for multiplex targets detection. **a** In the presence of a complementary target T3, P3 reacts with T3 to form a P3–T3 complex, which detaches from the GO surface, resulting in the fluorescence “on” state. **b** P1 assumes the fluorescence “off” state by the formation of probe–GO architecture, but switches to the “on” state by the interaction with thrombin. **c** In the presence of Ag^+^ or Hg^2+^, P4 or P5 is self-folded to form the stable C–Ag^+^–C or T–Hg^2+^–T structure, leading to the fluorescence “on” state. However, by continuing to add cysteine to the above solution, the C–Ag^+^–C or T–Hg^2+^–T structure is disrupted by Ag–S or Hg–S bond between cysteine and Ag^+^ or Hg^2+^, switching to the fluorescence “off” state again. Reprinted with permission from Ref. [135]. Copyright (2011) Elsevier

GO has a recombination of electron–hole pairs localized within *sp* ^2^ carbon clusters embedded within a *sp* ^3^ matrix and shows light absorption from UV to near-infrared (NIR) regions [131]. The largely dislocated *π*-electrons of GO lead to high fluorescence quenching ability which is very useful in optical-based biosensors [7, 132, 133]. Lu et al. demonstrated a biosensing platform based on GO for fluorescence-enhanced detection of target DNA. In their protocol, carboxyfluorescein (FAM)-labeled DNA (dye-labeled DNA) was noncovalently bound onto GO surface. The fluorescence of the dye was then completely quenched. In the presence of target DNA, the hybridization between dye-labeled DNA and target DNA disturbed the interaction between the dye-labeled DNA and GO. The dye-labeled DNA was released from GO, resulting in the restoration of fluorescence of dye. Fluorescence response of fluorescein-based dye-labeled aptamer–GO toward thrombin concentration was recorded. The limit of human thrombin detection was estimated to be about 2.0 nM [134]. Zhang et al. designed a versatile graphene-based fluorescence “on/off” switch for multiplex detection of various targets. In this work, GO, as a nanocarrier and dye quencher, was modified with ssDNAs labeled with different dyes to form a self-assembled ssDNA-graphene oxide (ssDNA-GO) architecture. In the presence of a target, competitive binding of the target and GO with the ssDNA resulted in the restoration of dye fluorescence. The sequence-specific DNA, protein (thrombin), metal ions (Ag^+^ and Hg^2+^), and a small molecule (cysteine) were applied to test the feasibility of simultaneously detecting different targets with this homogeneous ssDNA-GO architecture (Scheme 11). Different functional nucleic acids labeled with different dyes, such as oligonucleotide probes P1 (for thrombin), P3 (for DNA T3), P4 (for Ag^+^ and cysteine), and P5 (for Hg^2+^), were assembled onto the GO surface. After the incubation of the target mixture on the modified GO surface, fluorescence emission spectra of the mixture were recorded. One target can induce the disassembly of its corresponding functional ssDNA and result in an increase of fluorescence emission comparable to the fluorescence of the ssDNA-GO architecture. The detection limit was 1, 5, 20, 5.7, and 60 nM for sequence-specific DNA, thrombin, Ag^+^, Hg^2+^, and cysteine, respectively [135].

**Scheme 12:**
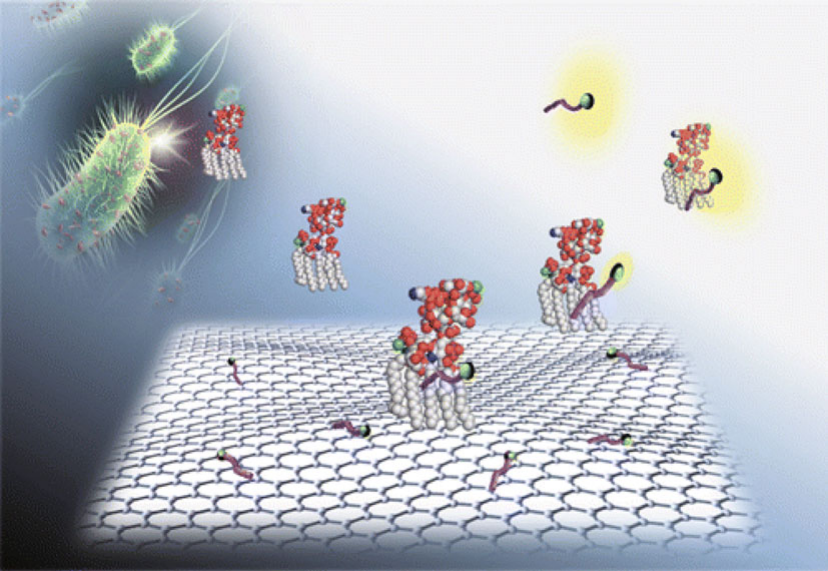
Schematic illustration of fluorescent turn-on sensor for lipopolysaccharide (LPS) detection using peptide-assembled GO. Reprinted with permission from Ref. [136]. Copyright (2015) American Chemical Society

A fluorescent turn-on sensor for lipopolysaccharide (LPS) detection using peptide-assembled GO was developed by Kim et al. LPS, released from the outer cell membrane of Gram-negative bacteria, is a toxic inflammatory stimulator. In this work, tetramethylrhodamine-labeled LPS-binding peptides were grafted on the GO surface. The fluorescence of the dye-labeled peptide was then quenched due to the interaction with GO. The bond between LPS and LPS-binding peptides led to the release of dye from GO and resulted in the fluorescence recovery (Scheme 12). The detection limit was found to be 130 pM [136].

**Scheme 13:**
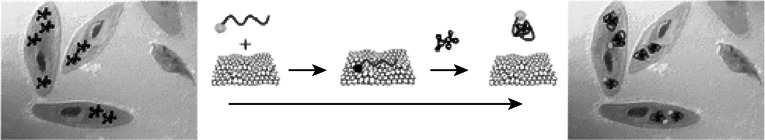
Schematic illustration of in situ molecular probing in living cells by using aptamer/GO-nS nanocomplex. Reprinted with permission from Ref. [137]. Copyright (2010) American Chemical Society

In order to investigate the sensing capabilities of graphene oxide nanosheets (GO-nS) in living cells, Wang et al. developed an aptamer/graphene oxide nanocomplex for intracellular monitoring and in situ molecular probing. As shown in Scheme 13, GO-nS were first modified with adenosine triphosphate (ATP) aptamer labeled with FAM and then incubated with mice epithelial cells (JB6 Cl 41-5a) in growth media. After the incubation, ATP aptamer was loaded and targeted with cellular ATP to form aptamer–ATP duplex and resulted in strong fluorescence recovery. GO-nS protected DNA well from enzymatic cleavage during the delivery and acted as a sensing platform with high fluorescence quenching efficiency for in vivo monitoring. Go was like an efficient cargo for cellular delivery of genes [137].

Graphene also can be used as SERS substrate to enhance Raman signals of absorbed biomolecules. He et al. reported a graphene-based SERS platform for multiplex DNA detection. Dye-labeled DNAs were assembled on gold-decorated graphene-SiO_2_/Si substrate. Due to the double effect of gold nanoparticles and graphene, the Raman signals of dye were dramatically enhanced. This platform exhibited extraordinarily high sensitivity and a detection limit of 10 pM for DNA. Moreover, it is possible to detect simultaneously more types of target DNAs on the proposed substrate with single-laser excitation [138].

Graphene has been also utilized to fabricate electrogenerated chemiluminescence (ECL) immunosensor. Xu et al. reported a graphene-based sandwich-type ECL biosensor for the detection of cancer marker prostate-specific antigen (PSA). The cathodic ECL behavior of luminol at a positive potential with a strong light emission on a graphene-modified electrode was presented in this work. A signal amplification strategy from enzyme–antibody-conjugated gold nanorods was developed. The graphene–chitosan-modified glassy carbon electrode was prepared for the immobilization of PAS capture antibodies to immobilize PSA. The gold nanorods were used to carry secondary antibody (Ab_2_) and GOx, which further amplified the ECL signal of luminol in the presence of glucose and oxygen. A linear relationship between ECL signals and the concentrations of PSA was obtained in the range from 10 pg mL^−1^ to 8 ng mL^−1^. The detection limit of PSA was 8 pg mL^−1^ [139].

## Conclusions and Perspectives

Compared to CNTs, graphene has a much younger history. With the development of graphene in biological and biomedical areas in recent years, a natural question came up is that which one is better for making biosensors. As two popular carbon nanomaterials, CNTs and graphene share some properties in common, including excellent mechanic, electronic, and thermal properties. Graphene can be massively produced at a low cost and does not have metallic impurities which often present in CNTs [9]. Graphene is much more amenable in large-scale architectures than high aspect ratio, one-dimensional CNTs. Due to the defect-rich property, graphene can be easily functionalized chemically to generate functional groups on the 2D plane. These advantages provide graphene more opportunities in biomedical imaging and therapy applications and offer more sensitive response as well [140]. In addition to the 2D nature, graphene has high electron mobility, making it a promising material in electronic biosensors. Graphene shows light absorption from UV to NIR regions and efficient fluorescence quenching properties. However, graphene does not have different chiral forms; thus, some optical properties caused by different chiral forms are unique for CNTs [87]. In applications in selective probing and imaging of living cells, graphene may need external labels, while CNTs do not need these.

Currently, it is a worldwide interest in employing carbon nanomaterials like CNTs and graphene in biosensors. It has been demonstrated by researchers that CNTs and graphene can offer new sensing protocols and also improve the detection limit over the existing protocols. The sensors using CNTs and/or graphene are faster in response, cheaper in cost, and smaller in size. However, great challenges remained in biosensors with CNTs and graphene. First, both CNTs and graphene need surface functionalization. The surface properties determine the subsequent performance in sensors. The established chemical approaches for surface functionalization are still far from being applicable and highly reproducible. The reliable and simple methods have to be developed for functionalizing CNTs and graphene in future. Second, biosensors are a class of devices that are able to translate the biological information to other information, like electric and optical signals. The integration of the sensing bed with the signal translator and receiver is another factor determining the efficiency of the sensor. The translation must be fast, reliable, and readable. Third, detecting more than one target molecules is still a great challenge in biosensor development. The medical devices usually require multifunctionality. Fourth, the sensors with strong anti-interference ability are highly desired. The real bioenvironment is much more complicated than the laboratory-built environment. Various biomolecules with similar properties often interfere with the detection of target molecules. To overcome this challenge, the effort must be made on developing highly specific targeting agents and these agents must be robust in various bioenvironments. To solve this problem and to develop CNT and graphene-based biosensors toward a practical application, efforts have to be made in not only materials science and technology, but also electric engineering and medical science.
